# Audiovisual Distraction Increases Prefrontal Cortical Neuronal Activity and Impairs Attentional Performance in the Rat

**DOI:** 10.1177/1179069517703080

**Published:** 2017-04-06

**Authors:** Douglas G Ririe, MD Boada, Benjamin S Schmidt, Salem J Martin, Susy A Kim, Thomas J Martin

**Affiliations:** Department of Anesthesiology, Wake Forest School of Medicine, Winston-Salem, NC, USA

**Keywords:** Attention, prefrontal, cortex, distraction, 5 choice, local field potential, electrophysiology, neuron spike, action potentials, whole animal, brain

## Abstract

Involvement of attentional processes is generally evidenced by disruption of behavior in the presence of distracting stimuli. The medial prefrontal cortex (mPFC) seems to play a role in fine-tuning activity during attentional tasks. A novel titration variant of the 5-choice serial reaction time task (5-choice serial reaction time titration variant [5CTV]) that adjusts task difficulty based on subject performance was used to evaluate the effects of audiovisual distraction (DSTR) on performance and mPFC single spike activity and local field potential (LFP). Attention was impaired in the 5CTV from DSTR, and mPFC spike activity was increased, whereas LFP was reduced. The increased spike activity in the mPFC in conjunction with DSTR suggests that conflicting attentional demands may contribute to the reduced task performance. As both hypo- and hyperactivation of the mPFC may contribute to attentional disruption, further studies using the 5CTV are needed to understand mPFC activity changes in real time during disruption of performance by other types of behavioral or neurobiological manipulations.

## Introduction

The brain is an extraordinarily complex organ that is commonly oversimplified from the perspective of its main function in any action or behavior. One view is that the brain’s operations are mainly intrinsic involving the acquisition and maintenance of information for interpreting, responding, and predicting environmental demands.^1,2^ The alternative concept exists that the brain is mainly reflexive, driven by the momentary demands of the environment.^3^ Unfortunately, combining controlled stimulation with carefully designed tasks (reflexive component) while simultaneously evaluating the behavioral relevance of electrical activity and its modulation (intrinsic component) has been extremely difficult to achieve. Nevertheless, behavioral and cognitive processes such as “attention” (defined as the selective focus of brain resources on a discrete aspect of information)^4^ remain relevant for understanding neurochemistry, pharmacology, and neurophysiology of brain circuitry.

Measurement of visual attention processing and impulse control in laboratory animals has been performed using the 5-choice serial reaction time task (5CSRTT).^5^ The behavioral measures are likely relevant to both attention-related disorders and mild cognitive dysfunction. A novel titration paradigm to the standard 5CSRTT method, the 5-choice serial reaction time titration variant (5CTV), was recently introduced.^6^ The titration variant (TV) of the 5C permits dynamic measurement of attention threshold across a wide range of performance and significantly decreases the time required for training.

The 5CTV remains a continuous performance test in the time and space domains and additionally has the sequential change in difficulty that is dependent on the outcome of each successive trial. The change in difficulty requires increasing allocation of resources to the task after each correct response to succeed in the next trial. This behavior is sensitive to visual distraction, similar to effects reported previously and in the classical 5CSRTT.^5–7^ However, the 5CTV permits the subject to find the level of sustained maximal performance in an automated manner as measured by the titrated cue duration (CD) over many trials. This allows the evaluation and quantification of impairment in maximal performance during a single session (within session) over time as well as between sessions over many trials. Despite the added feature of measuring maximal performance, the ability is preserved to evaluate the discrete measures of behavioral control, including accuracy of discrimination, impulsivity, perseverative responses, and response latencies.

Attention is an important component of many cognitive and executive processes. The medial prefrontal cortex (mPFC) plays a role in executive function in the rat.^8^ A component of attentional processing is in part regulated through mPFC activation to maintain maximal performance through a balance of activity and interactions with other brain regions.^8,9^ Dysfunctional attentional processing may occur as a result of either hypo- or hyperactivation in the mPFC.^10^ Regionally restricted neuronal electrical activity and its modulation by different interventions are important adjuncts in understanding the brain-related changes in neuronal activation that may modulate the behavioral outputs. Although numerous neurotransmitter systems play roles in attentional processing, cholinergic and noradrenergic inputs to the mPFC seem to play a role in maintaining discrimination from interference.^9,11^ Furthermore, neuronal activity in the mPFC has been shown to be predominantly increased with distraction.^11^ In this study, the aim is to measure the disruption from audiovisual distraction and evaluate the impact and possible role of mPFC activity changes in altered performance. We hypothesized that performance in the 5CTV would be correlated with the mPFC electrical activity in real time, and that presentation of distracting irrelevant audiovisual stimuli would impair attentional performance and produce discrete real-time neuronal activity changes in the mPFC in the freely behaving rat.

## Materials and Methods

### Animals

Male, Fischer 344 rats (N = 8, 240-350 g; Harlan Laboratories, Indianapolis, IN, USA) were used and kept on a reversed light:dark cycle (dark: 05:00-17:00). Animals were housed in a temperature-controlled and humidity-controlled room within an Association for Assessment and Accreditation of Laboratory Animal Care (AAALAC)-accredited facility as previously described.^6^ Briefly, after 1-week acclimation period with rats housed in pairs and given free access to standard rat chow and water, they were singly housed and given ad lib access to rat chow until they attained a minimum body weight of 240 g. Animals were then reduced to 90% of their free-feeding weight and given sufficient rat chow thereafter to maintain normal growth and increased weight gain while maintaining 90% of average free-feeding weight for Fischer 344 rats based on growth curves from the vendor. Animals were given free access to water except during experimental sessions. All procedures were in accordance with the Guide for the Care and Use of Laboratory Animals as adopted and promulgated by the National Institutes of Health and were approved by the Institutional Animal Care and Use Committee of Wake Forest School of Medicine (Winston-Salem, NC, USA). No animal was excluded from the study.

### Apparatus

All procedures were conducted in commercially available operant chambers controlled through a computer and interface using Med-PC IV software (Med Associates Inc., St. Albans, VT, USA). Operant chambers contained 1 wall with 5 nose poke holes with lights located in the rear of each and an illuminated food trough with infrared head entry detection located on the opposite wall with a pellet dispenser. The food trough lamp cap was replaced with a jeweled red lens cap (Allied Electronics Inc., Fort Worth, TX, USA). At the top of the wall with the food trough, a standard stimulus lamp with a red lens cap (house light) and an adjustable sonalert tone generator were placed (Med Associates Inc.). Chambers contained a standard stainless-steel grid bar floor, and each was contained within an expanded poly(vinyl chloride) sound and light attenuating cubicle (Med Associates Inc.).

### 5-choice titration variant training and behavior

All experiments were conducted during the dark phase of the light:dark cycle and once body weight stabilized at 90% of free-feeding weight as previously described.^6^ In phase 1, animals were trained to nose poke in the food trough where a lamp was illuminated to indicate 45-mg chocolate-flavored purified rat chow pellet availability (Bio-Serv Inc., Flemington, NJ, USA). Each successful nose poke was reinforced by delivery of 1 pellet and accompanied by a 0.5-second tone and turning off the food trough lamp for 0.5 second. Sessions lasted for 30 minutes or until the animal obtained 100 pellets, whichever occurred first. Once animals obtained 100 pellets for a minimum of 2 consecutive sessions, they graduated to the next phase of training.

In phase 2, the animals were trained to nose poke in the middle of the 5 nose poke holes located on the wall opposite to the food trough for food pellet reward. Sessions were initiated by delivery of 2 pellets into the food trough and illumination of the food trough light. Once the animal interrupted the head entry detector on the food trough, the trough lamp was turned off after 2 seconds and the trials were initiated, signaled by illumination of the house light. Each trial consisted of the light in the middle nose poke being illuminated for 30 seconds (30-second CD), during which time a nose poke resulted in the light being turned off and the food trough light being illuminated and delivery of 2 food pellets. Head entry detection at the food trough initiated a 2-second reward cycle timer, after which the food trough lamp was turned off and an intertrial interval (ITI) timer of 5 seconds was initiated. After the ITI, the next trial began, signaled by the illumination of the middle nose poke light. If the animal responded in a nose poke other than the middle one (incorrect response) or did not respond within 30 seconds (limited hold [LH], omission of response), the light was turned off and a 2-second time-out period was initiated during which all lights were turned off. Responses in any of the nose pokes during this time-out period reset the 2-second time-out timer. At the end of the time-out, the next trial was initiated signaled by illumination of the house light and, after the ITI, illumination of the middle nose poke light. Responses during the ITI were recorded as premature responses and resulted in initiation of a time-out. Sessions consisted of 50 trials or 30 minutes, whichever came first. Animals were required to complete all 50 trials with a minimum of 80% correct responses for 3 consecutive sessions before graduating to the third phase of training.

Phase 3 was identical to the second phase, with the exception that 1 of the 5 nose pokes was illuminated at random for each trial. The CD and LH were kept at 30 seconds, the ITI at 5 seconds, and the time-out and reward cycle at 2 seconds during this phase of training, and all other details of the procedure were identical to the second training phase. Animals were required to complete all 50 trials with a minimum of 80% correct responses before graduating to the final titration phase.

In phase 4, the TV paradigm of the 5-choice serial reaction time task (5CTV) was introduced and training was identical to the third phase of training, except that the CD and LH were altered based on the outcome of each trial. Each session consisted of 100 trials or 30 minutes, whichever came first. The CD was altered according to the series 30, 25, 20, 15, 10, 8, 6, 4, 2, 1, 0.8, 0.7, 0.6, 0.5, 0.4, 0.3, 0.2, and 0.1 seconds. The LH was set to the CD or 5 seconds, whichever was greater (ie, the animal always had a minimum of 5 seconds to respond or the entire CD if greater than 5 seconds). The CD was initially set to 30 seconds. If the animal made a correct response, the CD was decreased to the next lowest value in the series for the subsequent trial. If the animal made an incorrect response or an omission, the CD was increased to the next highest value in the series for the subsequent trial. If the animal made an incorrect response or omission when the CD was 30 seconds, or made a correct response when the CD was 0.1 second, the CD was not altered for the subsequent trial. Because of the titration element, there is a generalized balance between correct responses and omissions or incorrect responses to permit the determination of the maximal performance via an up-down method as previously described.^6^ This is quite different from the classical 5CSRTT paradigm. The median cue duration (MCD) was calculated from trials 16 to 100 (excluding the first 15 trials during which animals were titrating down the CD). Once the MCD was stable and titrated to <1-second duration for a minimum of 5 consecutive sessions (100 trials per session and the MCD calculated from trials 15 to 100), the animal was considered fully trained, and further surgery involving implantation of electrodes was performed.

### Experimental manipulation audiovisual distraction protocol

**Figure 1.**
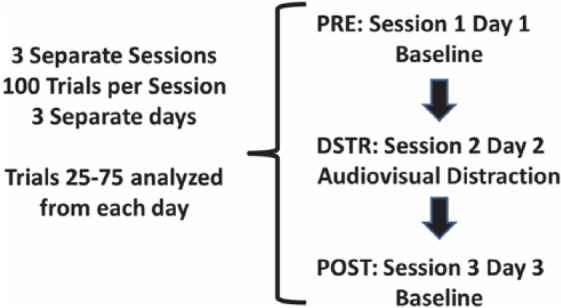
Study design. All animals underwent 100 trial sessions on consecutive days. After the performance was stable with a median cue duration (MCD) <1 second for a minimum of 1 week, the protocol began: PRE: baseline session on day 1, DSTR (distraction): during the audiovisual distraction session day 2, and POST: session on day 3 with no DSTR. DSTR occurred during trials 25 to 75. MCDs were determined for the same trial period as the DSTR (25-75). Single spike data are from the trials 25 to 75 and local field potential data are from a 300-second epoch during the trials 25 to 75.

A white stimulus lamp (Med Associates Inc.) was placed in the center of the Plexiglas chamber top. The lamp was illuminated at approximately 3 Hz (alternating 0.16 second ON, 0.16 second OFF) during trials 25 to 75 of individual sessions. The tone generator (2.9 kHz) was used to simultaneously create an audio distraction of 89 dB and was synchronized with the light distraction stimulus and delivered during the same trial period. Effects of DSTR on performance in the 5CTV were determined from trials 25 to 75 of 3 separate sessions on 3 separate days—PRE: baseline session day 1 with no DSTR, DSTR: during the audiovisual distraction session day 2, and POST: session on day 3 with no DSTR (Figure 1). All parameters for performance in the 5CTV were determined during the trials 25 to 75 over the 3 days and compared. This was similar to the electrophysiology recordings as described.

### Surgical implantation of electrodes

For in vivo field potential and spike recording, rats were allowed to free feed for 1 week after full training in the 5CTV. Rats were initially anesthetized with pentobarbital sodium 40 mg/kg, intraperitoneally, and maintained with oxygen and isoflurane. Animal breathing, reflexes, and level of anesthesia were monitored throughout the duration of the surgery. The animals were fixed in a stereotactic apparatus for implantation of recording electrodes in the mPFC. After sterile preparation with betadine and alcohol, the skin was incised and a small burr hole was made at the location identified by the stereotactic coordinates located from the bregma +2 mm anteriorly, 0.8 mm from midline on the right, and electrodes were then placed through the burr hole −3.5 mm from the bone surface. After placement, the electrodes were bent at the skull and fixed in dental cement mounted around stainless-steel mounting screws to provide further attachment and stabilization of the electrode. The posterior attachment screw formed the silver wire reference/ground electrode connection to the electrodes. The wireless electrodes were manufactured with 5 Teflon-coated 50 µm separate stainless-steel insulated electrodes (9-10 MΩ) (NB Labs, Denison, TX, USA) with the recording and ground electrodes attached to a 10-pin connector (Omnetics Connector Corporation, Minneapolis, MN, USA) and implanted with the connector facing upward.^12^ Care was taken to provide a smooth cement surface along the base of the implant so the skin could heal around the implant. After surgery, rats were given an intramuscular injection of 300 000 units/kg penicillin G to prevent infections, placed inside a heated cage to recover, and free fed for 1 week. Animals then began daily session of the 5CTV until the MCD stabilized for 5 consecutive days < 1 second. Animals then entered into the study protocol (Figure 1).

### Neural recording

A wireless headstage was connected to the implanted electrodes of each animal prior to each session and used to record, amplify, and digitize electrical activity from each electrode using NeuroWare acquisition software (Triangle BioSystems, Inc., Durham, NC, USA) for recording of extracellular local spike activity and local field potential (LFP). Digital online electrophysiological data are amplified and filtered to only record spike waveforms >2.5 times baseline signal/noise using negative and positive threshold and spike template, whereas the analog data are passed through a 1401 CED A-D converter (CED, Inc., Cambridge, UK). A low-pass filter was used for LFP <475 Hz, whereas a high-pass filter was used for spikes (300-7000 Hz), and a bandwidth filter was used (55-65 Hz) to reduce interference. One of the electrodes was selected as the reference electrode to eliminate muscle activity interference from chewing and licking. For each animal, thresholds for each electrode were determined over 5 baseline sessions, and the thresholds and reference electrodes were maintained for each electrode for each given animal over the experimental protocol. The effects of DSTR on neuronal firing and LFP were determined from trials 25 to 75 of 3 separate sessions on 3 separate days: PRE: baseline session day 1 with no DSTR, DSTR: during the audiovisual distraction session day 2, and POST: session on day 3 with no DSTR (Figure 1). Background electrophysiological data were determined with the animal in the operant chamber during the same time period with no stimuli present (no 5CTV). Single-cell spikes and LFP were recorded simultaneously from all animals. Local field potential was analyzed during a fixed window length of 300 seconds starting 60 seconds after the DSTR began. Electrode placement in the mPFC was verified with brain serial coronal cryosection at the conclusion of all experiments and compared with standard rat brain diagrams.

### Neural activity analysis

Offline analysis for further spike sorting was used for spike morphology to limit analysis to single-cell depolarizations which were standardized and quality controlled for consistent and reproducible analytics for the primary outcomes of absolute spike count, frequency and burst count, and frequency. Secondary analysis included maximum instantaneous frequency (IF), burst-related IF, and spike burst and frequency in bursts. NeuroExplorer version 4 (NEX Technologies, Madison, AL, USA) was used for analysis of LFP and spike characteristics. The term “burst” is a cluster of spikes from a single neuron that differs from other spikes by being more closely spaced in time than neighboring spikes thus having a higher discharge rate than the surrounding spike trains.^13^ Bursts were defined using the Poisson surprise method of Legéndy and Salcman.^14^ This method was implemented in NeuroExplorer and was used to quantify bursts between groups. The effects of distraction on LFP were evaluated using fast Fourier transform and comparing the total relative spectral power (in decibels or log of the power) over the 1 to 100 Hz range for a 300-second interval at the same time period during each of the 3 sessions during the trials 25 to 75.

### Data analysis/statistics

The primary outcome measure related to attention was the MCD that was calculated using Microsoft Excel from the CDs for trials 25 to 75 for each session. The effect of audiovisual distraction on MCD was analyzed using the Friedman repeated measures analysis of variance (ANOVA) on ranks, as the data were not normally distributed, and the pairwise comparisons were done using the Student-Newman-Keuls Method. The effects of the audiovisual distraction on secondary behavioral outcomes from the 5CTV were analyzed using repeated measures ANOVA with pairwise comparisons using Holms-Sidak method for multiple comparisons. Spike, burst, and LFP analyses were performed using 1-way repeated measures ANOVA with pairwise comparisons using the Holms-Sidak method for multiple comparisons. Statistical analysis was performed using SigmaPlot (Systat Software, Inc., San Jose, CA, USA). Data are presented as mean values and standard deviation except when not normally distributed, and then, median and range are presented and noted. By convention, *P* ⩽ .05 was considered statistically significant.

## Results

### Behavioral effects of DSTR in the 5CTV

**Figure 2.**
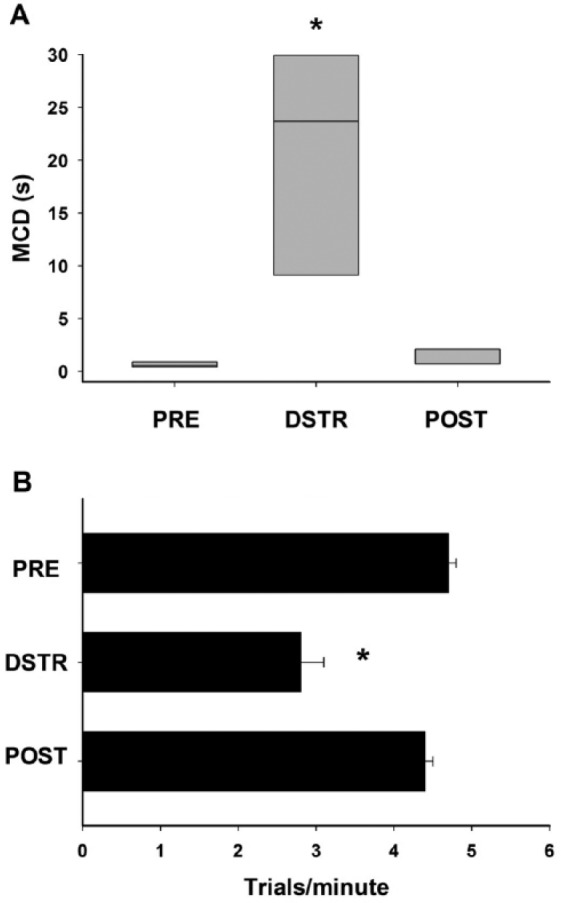
Distraction effect on MCD and speed of trials. Attentional performance was assessed using the MCD from trials 25 to 75. DSTR (distraction) produced a significant overall effect with an MCD that increased 40-fold compared with the PRE level. MCD returned to baseline in the POST session. Data are presented as median with the box representing the 25th to 75th percentiles. Rate of trial completion was also significantly changed by DSTR within session with DSTR decreasing the rate of trial completion compared with the PRE and POST sessions. Data are mean values with standard deviation. *P < .05 for pairwise comparison of DSTR compared with the PRE and POST sessions. (A) MCD and (B) trial completion rate. MCD indicates median cue duration.

**Figure 3.**
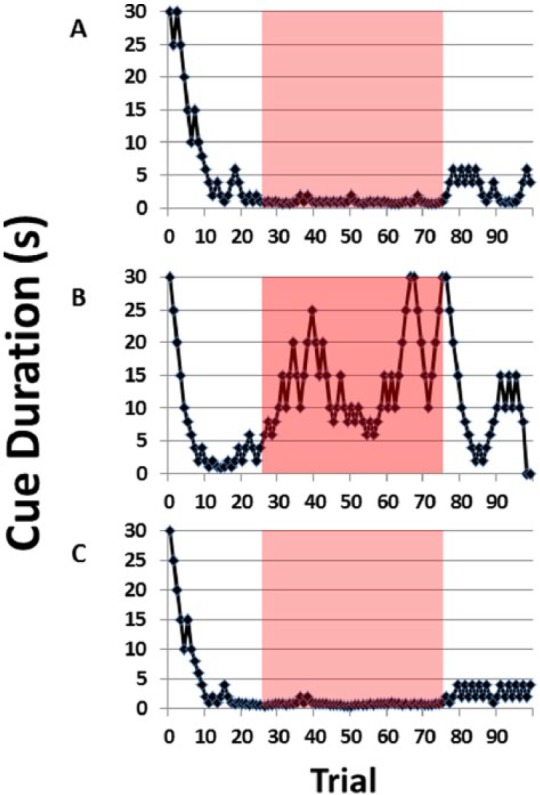
Changes in attentional performance from distraction. (A) The performance of a single representative animal is presented for PRE distraction, (B) during distraction (DSTR), and (C) POST distraction over all the trials within each session. This demonstrates the dynamic component of the 5-choice serial reaction time titration variant in measuring changes in performance. The pattern of performance is variable for each individual animal during the DSTR and this is the reason why the MCD is used to represent the performance over all the trials. The DSTR was present during trials 25 to 75 in the DSTR (B) only. The red highlighted areas represent the trials used for the MCD (trials 25-75). The corresponding MCDs for the animal from the 3 figures during the trials 25 to 75 were 0.8, 12.5, and 0.7 seconds for the PRE, DSTR, and POST sessions, respectively. The effect of the DSTR can be seen starting at trial 25 in the DSTR session, and at trial 75, the animal begins to be able to perform again. MCD indicates median cue duration.

The baseline (PRE) MCD, the measure of attentional performance in the 5CTV, was at median = 0.55 (range: 0.43-0.68) second and increased to median = 23.75 (range: 9.13-30.0) seconds during audiovisual DSTR, and this effect was largely resolved the next day (POST) with a return of the MCD to 0.7 (range: 0.7-1.88 seconds) (χ^2^(2) = 14.25, *P* < .001) (Figure 2A). All pairwise comparisons were significant (*P* < .05). The rate of completion of trials within the session decreased from PRE during DSTR and increased in the POST session (PRE (mean = 4.7, standard deviation = 0.1); DSTR (mean = 2.8, standard deviation = 0.8); POST (mean = 4.4, standard deviation = 0.4) (F_2,7_ = 48.51, *P* < .001) (Figure 2B). The performance of a single representative animal is presented PRE, during DSTR, and POST over all the trials within the session (Figure 3). This clearly demonstrates the dynamic component of the 5CTV in measuring changes in performance, the pattern of performance being variable for each individual animal and the reason why the MCD is used to represent the performance over all the trials. The mean number of correct, incorrect, and omitted responses PRE was as follows: mean = 56 standard deviation = 3, mean = 9 standard deviation = 5, and mean = 35 standard deviation = 6, respectively. The effects of DSTR are presented in Table 1. The number of correct, incorrect, and omitted responses in the 5CTV is generally very stable over time and between sessions. However, during DSTR, the number of correct and incorrect responses decreased to mean = 3, standard deviation = 16 (*P* < .05) and mean = 1, standard deviation = 1 (*P* < .05), respectively, whereas the number of omissions increased to mean = 46, standard deviation = 3 (*P* < .05). DSTR decreased the number of premature responses, but not perseverative responses (mean = 7, standard deviation = 2 PRE and mean = 1, standard deviation = 1 during DSTR; mean = 7, standard deviation = 3 PRE and mean = 4, standard deviation = 1 DSTR, respectively). Average latency for correct, incorrect, and reward retrieval responses PRE were as follows: mean = 1.3, standard deviation = 0.44; mean = 2, standard deviation = 0.9; and mean = 1.8, standard deviation = 0.2 second, respectively. DSTR increased the latency for correct responses only.

**Table 1.** Characteristics of the 5 choice titration variant performance during sessions.

	Overall *P* value	PRE	DSTR	POST
Correct	F_2,7_ = 14.85, *P* < .001	56 ± 3	35 ± 16*	54 ± 2
Omissions	F_2,7_ = 4, *P* < .001	35 ± 6	46 ± 3*	41 ± 7
Incorrect	F_2,7_ = 7.35, *P* = .007	9 ± 5	1 ± 1^†^	5 ± 5
Premature	F_2,7_ = 5.59, *P* = .016	7 ± 6	1 ± 2^†^	2 ± 2^‡^
Latency to correct	F_2,7_ = 13.08, *P* < .001	1.3 ± 0.44	4.0 ± 1.5*	2.3 ± 1.2
Latency to incorrect^§^	F_2,7_ = 2.13, *P* = .225	2.0 ± 0.9	5.8 ± 7.5	2.1 ± 1.5
Latency to reward^§^	F_2,7_ = 3.45, *P* = .06	1.8 ± 0.2	4.3 ± 3.5	2.1 ± 0.3

Statistical analysis was performed using 1 way repeated measures analysis of variance with pairwise comparisons using the Holms-Sidak method. PRE: day 1 baseline session, DSTR: day 2 audiovisual distraction session, POST: day 3 baseline session.

* *P* < .05 for DSTR versus PRE and POST and PRE and POST not different.

† *P* < .05 for PRE versus DSTR only.

‡ *P* < .05 for PRE versus POST only.

§ No significant differences.

### Single spike activity in the mPFC during 5CTV from DSTR

**Figure 4.**
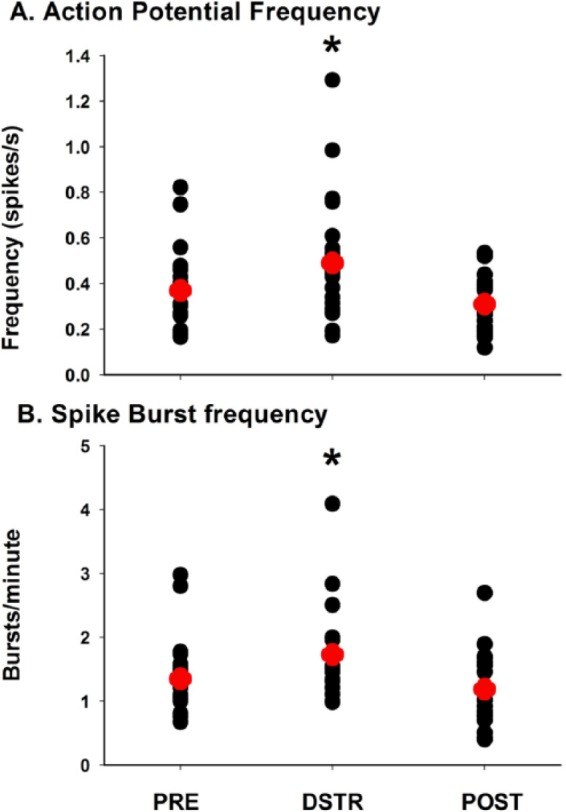
Effects of distraction on neuronal action potential spike and burst frequency. Spike and burst frequency during trials 25 to 75 of 3 separate sessions: PRE: session at baseline session day 1, DSTR (Distraction): during the audiovisual distraction session day 2 (DSTR), and POST: session on day 3 with no DSTR. Neuronal spike and burst analyses were performed from 21 distinct neuronal spike waveforms isolated from 40 electrodes in 8 animals. (A) There was an overall effect of DSTR with an increase in neuronal spike frequency from DSTR that returned to baseline values POST. (B) The overall burst rate was also increased from DSTR. Spike bursts increased with DSTR and returned to baseline in the POST session. The red marker represents the mean. *P < .05 for pairwise comparison of DSTR compared with the PRE and the POST sessions.

Neuronal spike analysis was performed from 21 distinct neuronal spikes isolated from 40 electrodes in 8 animals. Background data were gathered with the animal in the chamber during the same time interval with no stimuli present (no 5CTV). Effects of DSTR were determined during 3 separate sessions during trials 25 to 75 as outlined in the “Materials and Methods” section and Figure 1. Background number of total spikes fired was mean = 111, standard deviation = 10 in the dark chamber and increased to mean = 253, standard deviation = 27 during the PRE session (*P* < .05). DSTR produced an overall effect on total spikes during trials 25 to 75 (PRE [mean = 253, standard deviation = 124], DSTR [mean = 556, standard deviation = 270], POST [mean = 239, standard deviation = 98] [F_2,20_ = 29.94, *P* < .001]). Total spike number was significantly greater during DSTR compared with both PRE and POST (*P* < .05) with no difference between PRE and POST spikes. The background mean frequency of spikes was mean = 0.16, standard deviation = 0.06 and increased to mean = 0.37, standard deviation = 0.03 during the PRE 5CTV (*P* < .05). An overall effect on spike frequency was seen during DSTR (F_2,20_ = 5.75, *P* = .006) with DSTR spike frequency increasing significantly during DSTR compared with PRE (*P* = .005) or POST (*P* = .04) (Figure 4A). The background total number of bursts was mean = 7, standard deviation: 1. Total number of bursts increased during the PRE 5CTV to mean = 16, standard deviation: 2 (*P* < .05). An overall effect of DSTR was seen on total number of bursts (PRE [mean = 16, standard deviation = 7], DSTR [mean = 30, standard deviation = 12], POST [mean = 15, standard deviation = 8] [F_2,20_ = 27.23, *P* < .001]) with total burst during DSTR greater than PRE (*P* < .001) or POST (*P* < .001). The background mean rate of bursts (bursts/minute) was mean = 0.34, standard deviation = 0.11 and increased to mean = 1.35, standard deviation = 0.59 during the PRE 5CTV (*P* < .05). An overall effect on burst rate was seen during DSTR (F_2,20_ = 5.75, *P* = .006) with DSTR burst rate increasing significantly during DSTR compared with PRE (*P* = .006) or POST (*P* = .049) (Figure 4B). The characteristics of the spikes in bursts are presented in Table 2. The background percentage of spikes in bursts was mean = 31, standard deviation = 1%. The percentage of total spike in bursts increased during the PRE 5CTV to mean = 55, standard deviation = 2% and increased to mean = 70, standard deviation = 1% during DSTR and was reduced mean = 54, standard deviation = 2% POST (*P* < .05) (Figure 4) (burst spiking characteristics Table 1). The mean duration of burst at baseline was mean = 0.42, standard deviation = 0.03 and increased significantly in the 5CTV PRE (*P* < .05). DSTR increased mean burst duration (*P* < .05), which remained elevated in the POST session. Background mean frequency in burst was mean = 36, standard deviation = 11 Hz and increased to mean = 71, standard deviation = 6 Hz during the PRE 5CTV. This was significantly different from the mean spike frequency overall of mean = 0.38, standard deviation = 0.04 Hz (*P* < .05). The background number of spikes in bursts was mean = 74, standard deviation = 8 and increased to mean = 147, standard deviation = 20 during the PRE 5CTV. DSTR nearly tripled the total number of spikes in bursts to mean = 403, standard deviation = 48 (*P* < .05), which returned to PRE levels during the POST session. No difference in mean frequency, inter-spike interval (ISI), or peak frequency in the burst was seen during DSTR.

**Table 2.** Characteristics of spikes in bursts during distraction protocol.

	Overall *P* value	PRE	DSTR	POST
% spikes in bursts	F_2,20_ = 36.86, *P* < .001	55 ± 2	70 ± 1*	54 ± 2
Mean ISI	F_2,20_ = 4, *P* = .026	0.09 ± 0.011	0.08 ± 0.015	0.13 ± 0.024^†^
Mean frequency	F_2,20_ = 3.98, *P* = .026	71 ± 6	78 ± 6	55 ± 8^†^
Mean burst duration	F_2,20_ = 4.18, *P* = .022	0.72 ± 0.07^‡^	0.99 ± 0.13	1.17 ± 0.13
Mean spikes in bursts	F_2,20_ = 11.31, *P* < .001	9 ± 0.7	13 ± 0.8*	9 ± 0.5
Mean peak frequency^§^	F_2,20_ = 2.13, *P* = .13	419 ± 15	448 ± 10	406 ± 17
Total spikes in bursts	F_2,20_ = 33.19, *P* < .001	147 ± 20	403 ± 48*	132 ± 12

Abbreviation: ISI, inter-spike interval.

Statistical analysis was performed using 1 way repeated measures analysis of variance with pairwise comparisons using the Holms-Sidak method. PRE: day 1 baseline session, DSTR: day 2 audiovisual distraction session, POST: day 3 baseline session.

* *P* < .05 for DSTR versus PRE and POST and PRE and POST not different.

† *P* < .05 for POST versus DSTR only.

‡ *P* < .05 for PRE versus POST only.

§ No significant differences.

### LFP activity changes in the mPFC during 5CTV and DSTR

**Figure 5.**
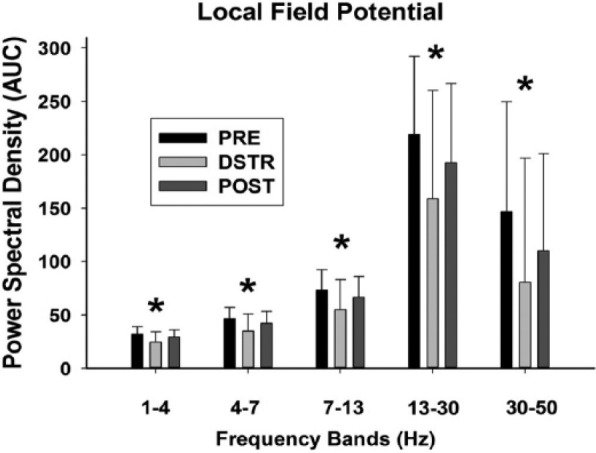
Effects of distraction on local field potentials. The power spectral density is the log of the frequency domain of the local field potentials in the medial prefrontal cortex and was determined from 21 electrode from the animals (N = 8) for a 300-second epoch (starting 60 seconds after the 25th trial) during trials 25 to 75 of 3 separate sessions: PRE: baseline session day 1, DSTR (distraction): during the audiovisual distraction session day 2, and POST: session on day 3 with no DSTR. Each bar represents the mean area under the power spectral density curve with standard deviation for each of the 5 noted frequency bands (delta [1-4 Hz], theta [4-7 Hz], alpha [7-13 Hz], beta [13-30 Hz], and low gamma [30-50 Hz] frequencies). DSTR decreased the overall power spectral density at each of the bands in the 0 to 50 Hz range (*P < .05). In the POST session, the power spectral density was not different from baseline.

**Figure 6.**
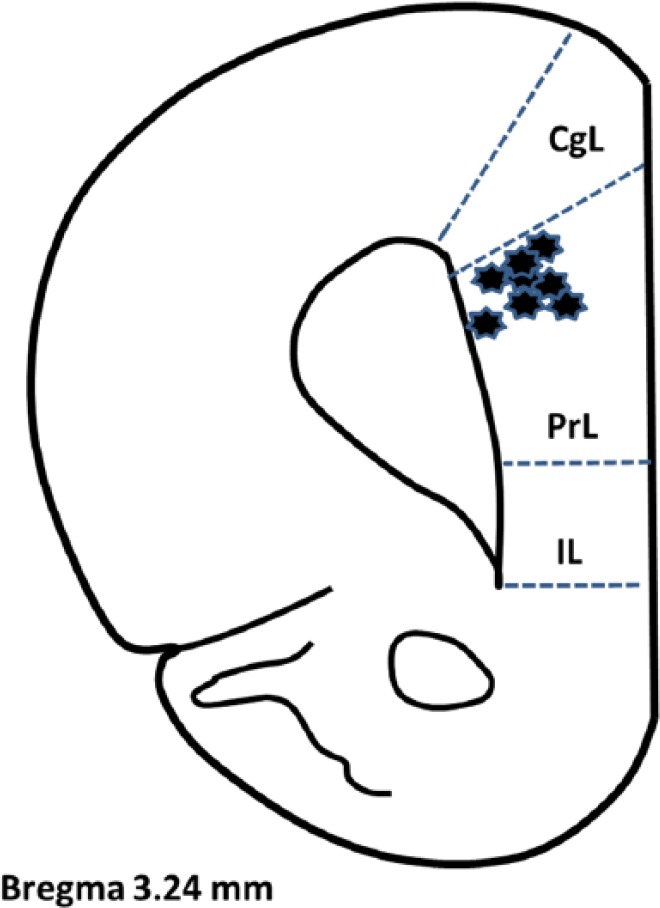
Location of recording electrode tips in the brain. Location of the tips of the recording electrodes in the PrL area of the medial prefrontal cortex verified at the conclusion of the experiments. All electrodes were placed on the right side. Coronal section 3.24 mm from Bregma according to Paxinos and Watson 2005. CgL indicates cingulate cortex; IL, infralimbic cortex; PrL, prelimbic.

The effects of DSTR on LFP were evaluated from the same electrodes as the spikes in each of the 8 animals using fast Fourier transform and comparing the total relative spectral power (in dB or log of the power) over the 1 to 100 Hz range for a 300-second interval at the same time period during each of the 3 sessions during the 25 to 75 trials period. Overall, there was an effect of DSTR on LFP with a reduction in area under the power spectral density curves from 0 to 50 Hz compared with the PRE session for all frequencies 1 to 50 Hz. Local field potential data are divided into 5 different frequency bands (delta [1-4 Hz] [F_2,20_ = 12.72, *P* < .001]; theta [4-7 Hz] [F_2,20_ = 12.40, *P* < .001]; alpha [7-13 Hz] [F_2,20_ = 10.57, *P* < .001]; beta [13-30 Hz] [F_2,20_ = 9.68, *P* < .001]; and low gamma [30-50 Hz] [F_2,20_ = 7.27, *P* = .002] frequencies). During the POST session, the power spectral density was not different from PRE values (Figure 5). The power spectral density was too low to be meaningful in the 50 to 100 Hz range. Placement of the electrodes was verified at the conclusion of the experiments and location shown in Figure 6.

## Discussion

These data corroborate the value of the 5CTV in assessing cognitive functional status with respect to attentional performance. In addition, we have shown that audiovisual disruption of attentional performance can be readily assessed using the 5CTV. Measurement of neuronal activity in the mPFC suggests that increased activity above background (no 5CTV) occurs while performing the 5CTV attention task. In addition, the role of the neuronal activity in the mPFC regarding attentional performance and disruption is further established through changes in both individual spike activity and LFP power that correlate with changes in attentional performance in the 5CTV as measured by the MCD. The MCD therefore appears to be an accurate reflection of mPFC activity in real time during the 5CTV task, both at baseline and during disrupted performance.

In this study using the 5CTV, a decrease in correct and incorrect responses and an increase in omissions occurred. This is consistent with data in the standard 5C using a continuous flashing light as a distractor.^7^ Because of the titration element, there is a generalized balance between correct responses and omissions or incorrect responses to permit the determination of the maximal performance via an up-down method as delineated in the “Materials and Methods” section and as previously described.^6^ This is quite different from the classical 5CSRTT paradigm. Other studies of intermittent noise and light distraction have been done which also resulted in reduced correct responses.^15–17^ A significant or persistent distraction can increase demand for selective attention, which will either result in mitigating the distraction over time or in the animal disengaging from the task and redirecting attention toward the distraction. The extent and degree of the behavioral disruption from the DSTR in our study likely resulted in increased attention focused on the concern for the novel light and sound and its possible implications. The increased time latency for responding in behaviors in our study may reflect a conflict between attention processing of reinforcing stimuli and the attentional demand of unknown risks from novel stimuli (DSTR). Latency to retrieve food was not significantly increased, although an increase in reward retrieval may not represent decreased motivation for food per se, but rather prioritization of attention to potentially harmful stimuli from DSTR relative to known rewards (food). All effects from DSTR appear to be due to conflict from multiple inputs signaling to the mPFC simultaneously, as both the 5CTV and DSTR significantly increased mPFC spike activity in real time, necessitating that the animal prioritizes actions based on disparate inputs. In our study, premature responses were not increased by the distraction, but rather decreased. In previous studies using the standard 5C, premature responses were increased.^15^ The reason for the difference is unclear. In the classic 5C, it was suggested that increased premature responses may have been a result of disorientation from the distractor or nonspecific arousal leading to a less conservative response pattern. In our paradigm, this disorientation could contribute to delayed decision making or even to the increased omissions due to inability to prioritize the 5CTV stimuli, which seemed to occur in all behaviors. Light and sound may disrupt attention and performance differentially and could be partially responsible. Disruption in attention may occur differently through alternate perceptual dimensions; in this case, adding the audio (irrelevant modality) may disrupt attention differently and through different brain regions than a visual stimulus, visual stimuli being the reinforced sensory modality in the 5CTV rather than sound.^18^

The PFC is involved in higher order executive tasks such as working memory, learning, and behavioral flexibility. The mPFC was targeted in this study as it has been implicated in attentional processing, and measuring neuronal activity in this region allows further validation of the role of mPFC in attentional performance and processing in the 5CTV as measured through the MCD and its disruption with audiovisual DSTR.^7,8,15–17^ Bursting increased with DSTR in this study. In addition, the character of the bursts changed with increased burst duration and increased spikes within the burst. Bursting may be a generalized amplifying mechanism. Increased burst activity may be related to increased neurotransmitter release and information processing, whereas reduced burst activity may attenuate the signal transmission efficiency. Complicating the implications of bursting activity is that both increased and decreased bursting may result in reduced performance of behaviors.^19^ Neurons analyzed in the mPFC in this study were capable of bursting and single spike firing at the same time, similar to a subset of neurons reported previously.^20^ Despite the largely held belief that bursting serves to enhance communication to nerves, it is possible that certain neurons are not responsive to high-frequency stimulation.^21^ This alternative possibility may allow a burst to communicate with neurons in the circuit without affecting the usual suspects that are responsive to single depolarization; thus, a burst could shut off one cell and turn on another, likely different than the communication that occurs with single spikes. Single spikes may be used to dampen and may be associated with habituation to unimportant events. Thus, switching from single spike to bursts may allow neurons to conditionally code various aspects of a behavior and open lines of synaptic activity that are unresponsive to single spike or bypass other neurons that may be unresponsive to the high-frequency bursts. Thus, switching may permit selective communication with different neuronal subsets depending on the circumstances.

A generalized reduction in activity in the mPFC was noted as measured by LFP. This suggests reduced input to the mPFC during DSTR as LFP is thought to be dominated by input, whereas spike activity is more related to output activity of local neurons. Thus, decreased input to the mPFC may produce an increase in output of the mPFC if the predominate input is inhibitory and DSTR decreased inhibitory input. A number of factors may have contributed to the increased spike activity in the mPFC and reduced attentional performance. The audiovisual distraction was likely very stressful, and stress alone may increase firing rate in mPFC neurons related to effects via glucocorticoid signaling.^11,22,23^ Increased single spike and burst activity in the mPFC could be modulated through reduced inhibitory input effects at γ-aminobutyric acid.^24,25^ This could be possible with the reduced LFP seen during DSTR possibly suggesting a decrease in inhibitory input. Another factor may be that the 5CTV is a high-stimulus demand behavior because the cue is titrated to ability to perform. Cholinergic deafferented animals perform similarly under low stimulus demands, but under high attentional demand, reduced performance is seen.^26^ As animals are forced to perform at their limits in the 5CTV, decrements in sustained performance caused by DSTR in this paradigm likely involve disruption in cholinergic signaling. In the POST session, there could still be reduced input and neurotransmitter release from other regions, possibly due to reduced attention to the task in anticipation of the return of the DSTR. This is also consistent with the persistent mildly increased MCD in the POST session.

The 5CTV is a robust and unique paradigm being sensitive to disruption from audiovisual distraction. Because it involves attentional performance that is titrated to the shortest CD, understanding the role of different brain areas and pharmacology in the task is valuable. Although mPFC seems to play a role in task performance, further studies will need to involve trial-specific data analysis of neuronal activity to better understand the effects of the changes in activity in the mPFC. This will need to include spike activity changes for correct, incorrect, and omission trials, as well as LFP data, as disruption of neural activity in error trials has been shown.^27^ This more granular analysis will need to take into consideration the result of the prior trial to better understand the coding. Simultaneous recording from other locations may also help understand the roles of the mPFC in relation to attentional processing and performance in the 5CTV. In addition, our study only used male rats, and the role of biological sex in mPFC activation from disruption will be valuable to understanding sex-related differences in attentional performance.^28^ Although more work needs to be done, the rapid training paradigm, the establishment of sensitivity to disruption, and the role of the mPFC suggest that the 5CTV is a valuable tool for further probing attentional processing. With this in mind, the 5CTV should be an important adjunct for understanding the influences and implications of PFC function in the cause and treatment of illnesses related to attentional impairment.

## References

[1] Llinás RR I of the Vortex: From Neurons to Self. Cambridge, MA: MIT Press; 2001.

[2] Raichle ME Two views of brain function. Trends Cogn Sci. 2010;14:180–190. 20206576 10.1016/j.tics.2010.01.008

[3] Sherrington CS The Integrative Action of the Nervous System. New Haven, CT: Yale University Press; 1906.

[4] Anderson JR Cognitive Psychology and Its Implications. 6th ed. New York, NY: Worth Publishers; 2005.

[5] Bari A Dalley JW Robbins TW The application of the 5-choice serial reaction time task for the assessment of visual attentional processes and impulse control in rats. Nat Protoc. 2008;3:759–767. 18451784 10.1038/nprot.2008.41

[6] Martin TJ Grigg A Kim SA Ririe DG Eisenach JC Assessment of attention threshold in rats by titration of visual cue duration during the five choice serial reaction time task. J Neurosci Methods. 2015;241:37–43. 25528113 10.1016/j.jneumeth.2014.12.007 PMC4323678

[7] Amitai N Markou A Comparative effects of different test day challenges on performance in the 5-choice serial reaction time task. Behav Neurosci. 2011;125:764–774. 21942437 10.1037/a0024722 PMC3187548

[8] Dalley JW Cardinal RN Robbins TW Prefrontal executive and cognitive functions in rodents: neural and neurochemical substrates. Neurosci Biobehav Rev. 2004;28:771–784. 15555683 10.1016/j.neubiorev.2004.09.006

[9] Robbins TW The 5-choice serial reaction time task: behavioural pharmacology and functional neurochemistry. Psychopharmacology (Berl). 2002;163:362–380. 12373437 10.1007/s00213-002-1154-7

[10] Pezze M McGarrity S Mason R Fone KC Bast T Too little and too much: hypoactivation and disinhibition of medial prefrontal cortex cause attentional deficits. J Neurosci. 2014;34:7931–7946. 24899715 10.1523/JNEUROSCI.3450-13.2014 PMC4044251

[11] Gill TM Sarter M Givens B Sustained visual attention performance-associated prefrontal neuronal activity: evidence for cholinergic modulation. J Neurosci. 2000;20:4745–4757. 10844044 10.1523/JNEUROSCI.20-12-04745.2000 PMC6772472

[12] Nicolelis MA Lin RC Woodward DJ Chapin JK Dynamic and distributed properties of many-neuron ensembles in the ventral posterior medial thalamus of awake rats. Proc Natl Acad Sci U S A. 1993;90:2212–2216. 8460124 10.1073/pnas.90.6.2212 PMC46056

[13] Lobb C Abnormal bursting as a pathophysiological mechanism in Parkinson’s disease. Basal Ganglia. 2014;3:187–195. 24729952 10.1016/j.baga.2013.11.002 PMC3979569

[14] Legéndy CR Salcman M Bursts and recurrences of bursts in the spike trains of spontaneously active striate cortex neurons. J Neurophysiol. 1985;53:926–939. 3998798 10.1152/jn.1985.53.4.926

[15] Carli M Robbins TW Evenden JL Everitt BJ Effects of lesions to ascending noradrenergic neurones on performance of a 5-choice serial reaction task in rats; implications for theories of dorsal noradrenergic bundle function based on selective attention and arousal. Behav Brain Res. 1983;9:361–380. 6639741 10.1016/0166-4328(83)90138-9

[16] Hahn B Stolerman IP Nicotine-induced attentional enhancement in rats: effects of chronic exposure to nicotine. Neuropsychopharmacology. 2002;27:712–722. 12431846 10.1016/S0893-133X(02)00348-2

[17] Hahn B Shoaib M Stolerman IP Nicotine-induced enhancement of attention in the five-choice serial reaction time task: the influence of task demands. Psychopharmacology (Berl). 2002;162:129–137. 12110990 10.1007/s00213-002-1005-6

[18] Ng CW Noblejas MI Rodefer JS Smith CB Poremba A Double dissociation of attentional resources: prefrontal versus cingulate cortices. J Neurosci. 2007;27:12123–12131. 17989278 10.1523/JNEUROSCI.2745-07.2007 PMC6673242

[19] Jackson ME Homayoun H Moghaddam B NMDA receptor hypofunction produces concomitant firing rate potentiation and burst activity reduction in the prefrontal cortex. Proc Natl Acad Sci U S A. 2004;101:8467–8472. 15159546 10.1073/pnas.0308455101 PMC420417

[20] Cooper DC The significance of action potential bursting in the brain reward circuit. Neurochem Int. 2002;41:333–340. 12176075 10.1016/s0197-0186(02)00068-2

[21] Izhikevich EM Desai NS Walcott EC Hoppensteadt FC Bursts as a unit of neural information: selective communication via resonance. Trends Neurosci. 2003;26:161–167. 12591219 10.1016/S0166-2236(03)00034-1

[22] Devilbiss DM Jenison RL Berridge CW Stress-induced impairment of a working memory task: role of spiking rate and spiking history predicted discharge. PLoS Comput Biol. 2012;8:e1002681. 23028279 10.1371/journal.pcbi.1002681 PMC3441423

[23] Meng QY Chen XN Tong DL Zhou JN Stress and glucocorticoids regulated corticotropin releasing factor in rat prefrontal cortex. Mol Cell Endocrinol. 2011;342:54–63. 21664419 10.1016/j.mce.2011.05.035

[24] Wang GW Cai JX Disconnection of the hippocampal-prefrontal cortical circuits impairs spatial working memory performance in rats. Behav Brain Res. 2006;175:329–336. 17045348 10.1016/j.bbr.2006.09.002

[25] Asinof SK Paine TA Inhibition of GABA synthesis in the prefrontal cortex increases locomotor activity but does not affect attention in the 5-choice serial reaction time task. Neuropharmacology. 2013;65:39–47. 23022048 10.1016/j.neuropharm.2012.09.009 PMC3521100

[26] Ljubojevic V Luu P De Rosa E Cholinergic contributions to supramodal attentional processes in rats. J Neurosci. 2014;34:2264–2275. 24501365 10.1523/JNEUROSCI.1024-13.2014 PMC6608542

[27] Laubach M Caetano MS Narayanan NS Mistakes were made: neural mechanisms for the adaptive control of action initiation by the medial prefrontal cortex. J Physiol Paris. 2015;109:104–117. 25636373 10.1016/j.jphysparis.2014.12.001 PMC5292776

[28] Bayless DW Darling JS Stout WJ Daniel JM Sex differences in attentional processes in adult rats as measured by performance on the 5-choice serial reaction time task. Behav Brain Res. 2012;235:48–54. 22835820 10.1016/j.bbr.2012.07.028

